# Regeneration in pig livers by compensatory hyperplasia induces high levels of telomerase activity

**DOI:** 10.1186/1476-5926-6-6

**Published:** 2007-07-02

**Authors:** Henning Wege, Anett Müller, Lars Müller, Susan Petri, Jörg Petersen, Christian Hillert

**Affiliations:** 1 Department of Gastroenterology and Hepatology with Sections Infectious Disease and Tropical Medicine, University Medical Center Hamburg-Eppendorf, Martinistrasse 52, 20246 Hamburg, Germany; 2 Department of Hepatobiliary Surgery and Visceral Transplantation, University Medical Center Hamburg-Eppendorf, Martinistrasse 52, 20246 Hamburg, Germany; 3 Department of Pathology, University Medical Center Hamburg-Eppendorf, Martinistrasse 52, 20246 Hamburg, Germany

## Abstract

**Background:**

Several highly proliferative human cells transiently activate telomerase, a ribonucleoprotein with reverse transcriptase activity, to counterbalance replication-associated telomere erosion and to increase stress resistance. Quiescent human hepatocytes exhibit very low or undetectable levels of telomerase activity. However, hepatocytes display a remarkable proliferative capability following liver injury. To investigate whether liver regeneration by compensatory hyperplasia is associated with telomerase activation, we measured telomerase activity in pig livers after 70 to 80% partial hepatectomy using a fully quantitative real-time telomeric repeat amplification protocol. In contrast to commonly studied inbred laboratory mouse strains, telomere length and telomerase activity in porcine tissues are comparable to humans.

**Results:**

Following partial hepatectomy, histology revealed mitotic hepatocytes as marker for compensatory hyperplasia. As expected, there was no induction of inflammation. Telomerase activity increased significantly showing the highest levels (5-fold upregulation) in pigs treated with partial hepatectomy and hepatic decompression. Moreover, telomerase activity significantly correlated to the number of mitotic hepatocytes.

**Conclusion:**

Our data demonstrate telomerase activation in liver regeneration by compensatory hyperplasia in a large animal model with telomere biology comparable to humans. Telomerase activation may constitute a mechanism to protect proliferating liver cells against telomere shortening and oxidative stress.

## Background

The human liver possesses a remarkable capability to restore its functional capacity following liver injury by a process termed compensatory hyperplasia [1,2]. Differentiated and normally quiescent hepatocytes are the primary cell type responsible for liver regeneration, especially following partial hepatectomy or administration of carbon tetrachloride in rodent models [3]. As reserve compartment, bipotent hepatic progenitor cells are activated if extensive loss or damage of hepatocytes with an impaired replication capability occurs [4].

In most somatic human cells, cellular proliferation is associated with progressive telomere shortening. Telomeres are specialized high-order chromatin structures that protect chromosome ends against degradation by forming molecular caps. In addition to telomere-stabilizing proteins, telomeres consist of tens of kilo bases of telomeric repeats [5,6]. After a certain number of cell divisions, replication-associated telomere shortening renders telomeric caps unstable and chromosome ends unprotected. This results in a dramatic upsurge in chromosomal aberrations. Additionally, cells with unstable chromosome ends activate their DNA damage response machinery with entry into cell cycle exit and replicative senescence, a post-mitotic quiescent state [7]. In contrast to somatic cells, human germ and embryonic stem cells are capable of undergoing an infinite number of cell divisions [8] In these cells, the enzyme complex telomerase counterbalances telomere erosion by de novo synthesis of telomeric repeats onto chromosome ends [9]. Interestingly, telomerase activation in embryonic stem cells is also associated with increased resistance to differentiation- and stress-induced apoptosis [10,11].

Most differentiated human cells, including quiescent hepatocytes, have low or undetectable levels of telomerase activity [12]. However, certain highly proliferative human cell types, such as cells in the regenerative basal layer of epidermis [13] and B lymphocytes in the germinal center [14-16], transiently express high levels of telomerase activity upon commitment to clonal expansion. In line with this observation, recent data indicate that telomerase is actively regulated throughout the cell cycle in murine hepatocytes [17]. Unfortunately, there are significant differences between mice and humans regarding telomere biology that are possible concerns in the use of mouse models to investigate telomere maintenance and telomerase regulation. For example, in marked contrast to humans, commonly employed inbred laboratory mouse strains have approximately ten times longer telomeres (up to 150 kilo bases) and express robust levels of telomerase activity in a wide range of somatic tissues, including normal liver [18]. According to current studies, these divergences may be attributed to absence of a cis-acting element repressing *TERT *promoter activity in murine cells [19].

Pigs display no or very low levels of telomerase activity in the liver [20]. Moreover, pig telomeres are comparable to those of humans regarding length and shortening during aging [21,22]. Because of these similarities, pigs have been utilized as model system to investigate telomerase regulation and telomere dynamics in mammalians [23]. In a previous pig liver regeneration study, maximum regenerative response occurred three days after 70% partial hepatectomy [24]. The timing of the regenerative response in pig livers is therefore comparable to other large animal models with maximum mitotic activity on the 3rd postoperative day, which contrasts the regenerative peak observed in rats after only 24 h post liver injury. To test our hypothesis that liver regeneration by compensatory hyperplasia is associated with telomerase activation, we quantitated telomerase activity three days after partial hepatectomy in pig liver tissue samples. Furthermore, we correlated telomerase activity to the number of mitotic hepatocytes and the degree of inflammatory infiltration.

## Results

### Surgical procedures and clinical chemistry

All surgical procedures were performed without relevant perioperative mortality. As shown in Table 1, animals were evenly distributed between the three experimental groups (6 animals per group), and median weights were not significantly different. Three days after surgery, liver samples were collected and evaluated for mitotic activity, hepatitis score, and telomerase activity. Blood samples were obtained from all animals included in this investigation (n = 18) before and on each day after surgery. Clinical chemistry results are summarized in Table 2.

**Table 1:** Experimental groups and baseline characteristics.

**Group**	**N**	**Gender (male/female)**	**Body weight (kg)^1^**	**P^2^**
**LT**	6	2/4	33.8 [29.0 – 45.0]	
**PH**	6	2/4	33.8 [27.0 – 45.5]	1.000
**PH/TIPS**	6	5/1	29.5 [24.0 – 44.9]	0.240

^1^Weights expressed as median [range]. ^2^Comparison to LT with P < 0.05 (Mann-Whitney U test) considered significant. Abbreviations: LT, laparotomy; PH, partial hepatectomy; TIPS, transjugular intrahepatic portosystemic shunt.

**Table 2:** Clinical chemistry.

**Parameter**	**Hours**	**LT **(n = 6)	**PH **(n = 6)	**P^1^**	**PH/TIPS **(n = 6)	**P^1^**
**Albumin (g/l)**	0	39 [30 – 43]	40 [38 – 43]	0.485	41 [35 – 41]	0.818
	24	42 [33 – 43]	38 [35 – 39]	0.329	36 [31 – 39]	0.082
	48	39 [31 – 42]	39 [34 – 41]	0.931	36 [33 – 40]	0.429
	72	38 [28 – 40]	36 [30 – 39]	0.589	32 [28 – 35]	0.132
**Bilirubin (mg/dl)**	0	0.2 [0.2 – 0.2]	0.2 [0.2 – 0.2]	1.000	0.2 [0.2 – 0.2]	1.000
	24	0.2 [0.2 – 0.4]	0.7 [0.3 – 1.3]	0.009	1.1 [0.5 – 1.9]	0.004
	48	0.2 [0.2 – 0.4]	0.8 [0.4 – 1.5]	0.004	1.3 [0.3 – 5.7]	0.009
	72	0.2 [0.2 – 0.2]	0.6 [0.4 – 1.3]	0.002	1.7 [1.2 – 5.5]	0.002
**AST (U/l)**	0	21 [8 – 29]	13 [9 – 47]	0.537	16 [9 – 21]	0.310
	24	69 [38 – 200]	410 [271 – 685]	0.004	827 [206 – 1237]	0.004
	48	47 [33 – 147]	424 [157 – 860]	0.004	912 [729 – 2144]	0.004
	72	37 [21 – 94]	259 [102 – 642]	0.002	692 [363 – 1976]	0.002
**ALT (U/l)**	0	23 [18 – 47]	28 [18 – 67]	0.589	23 [18 – 37]	0.937
	24	33 [26 – 67]	87 [33 – 141]	0.030	90 [48 – 222]	0.017
	48	32 [23 – 55]	84 [32 – 174]	0.017	153 [72 – 1321]	0.004
	72	38 [17 – 48]	62 [28 – 108]	0.052	120 [71 – 207]	0.002

^1^Comparison to LT with P < 0.05 (Mann-Whitney U test) considered significant. Values expressed as median [range]. Abbreviations: ALT, alanine aminotransferase; AST, aspartate aminotransferase; LT, laparotomy; PH, partial hepatectomy; TIPS, transjugular intrahepatic portosystemic shunt.

As expected for animals receiving partial hepatectomy (PH) with and without transjugular intrahepatic portosystemic shunt (TIPS), a decrease in serum albumin was observed. Furthermore, a steady and significant increase in serum bilirubin was detected in PH and PH/TIPS animals. Changes in serum bilirubin were more pronounced in the PH/TIPS group with 6- to 8-fold higher values compared to animals with control laparotomy (LT) on the 2nd and 3rd postoperative day. In addition, clinical chemistry revealed a significant increase in serum aspartate aminotransferases (AST) and alanine aminotransferases (ALT) following PH and PH/TIPS. In PH and PH/TIPS animals, serum AST and ALT reached peak values on the 2nd postoperative day and began to drop on the 3rd postoperative day. Falling AST and ALT confirmed absence of prolonged postoperative liver injury. Other liver function parameters did not show consistent changes during the observation period. In summary, clinical chemistry in animals following PH or PH/TIPS reflected loss of functional liver parenchyma and surgical induction of liver injury with temporary rise of serum aminotransferases.

### Mitotic activity and hepatitis scores

**Figure 1:**
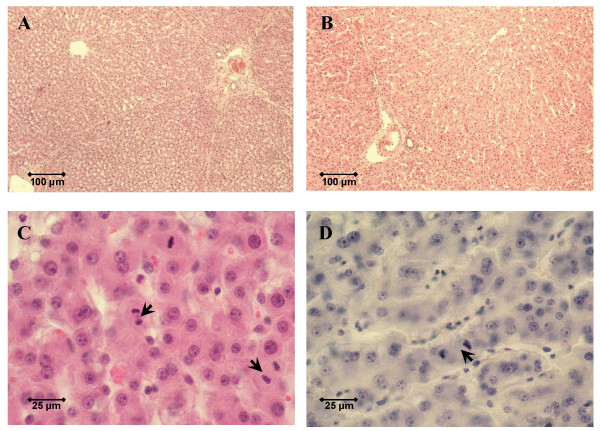
**Histology three days after surgery**. Histology of pig livers three days after surgery was assessed to grade liver regeneration and hepatitis. Shown are representative low-power fields (original magnification 100×) in hematoxylin and eosin-stained sections of a control pig liver without surgery (A) and a pig liver three days after 70 to 80% hepatectomy (B). High-power fields (original magnification 400×) were used to count the number of mitotic hepatocytes (arrows) per ten visual fields. Representative microphotographs are shown for a pig liver three days after 70 to 80% hepatectomy (C) and a pig liver three days after 70 to 80% hepatectomy with hepatic decompression (D).

**Figure 2:**
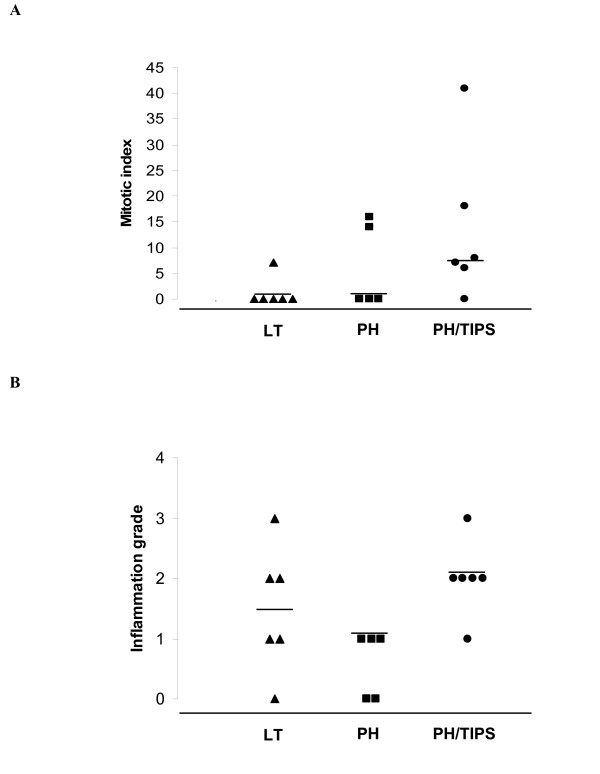
**Mitotic indices and hepatitis scores three days after surgery**. The dot plots depict distribution of mitotic indices (A) and hepatitis scores (B) determined in stained liver-sections of pigs on the 3rd postoperative day after control laparotomy (LT, n = 6) or partial hepatectomy (PH, n = 5) with and without transjugular intrahepatic portosystemic shunt (TIPS, n = 6). Each dot represents data for a single animal. Median values are marked by horizontal bars. Histological assessment is not available for one PH animal.

Mitotic hepatocytes were identified in the majority of liver samples on the 3rd postoperative day after PH or PH/TIPS (Figures 1C and 1D). The highest number of mitotic hepatocytes was detected in the PH/TIPS group (Figure 2A). All but one animal in the PH/TIPS group (n = 6) had detectable mitotic hepatocytes with a median mitotic index (MI), *i.e*., mitotic hepatocytes per ten high-power fields, of 7.5 and a maximum value of 41 (Figure 2A). In contrast, mitotic activity was observed in only one out of six animals three days after LT. Taken together, mitotic hepatocytes as direct marker for liver regeneration by compensatory hyperplasia were clearly identified in 65% of PH and PH/TIPS animals (n = 11; histological assessment not available for one PH animal).

As expected for liver resection models, histology did not show relevant induction of hepatitis by PH or PH/TIPS (Figure 2B). Only one LT and one PH/TIPS animal showed grade 3 hepatitis. Grade 4 hepatitis was not observed in this study. Moreover, although the median hepatitis score was slightly higher in the PH/TIPS group (2.0 [1-3], n = 6; median [range]) compared to animals with LT (1.5 [0 – 3], n = 6) or PH (1.0 [0 – 1], n = 5), these differences were not significant. In addition, animals treated by PH showed a lower median hepatitis score in comparison to LT animals.

### Telomerase activation

**Figure 3:**
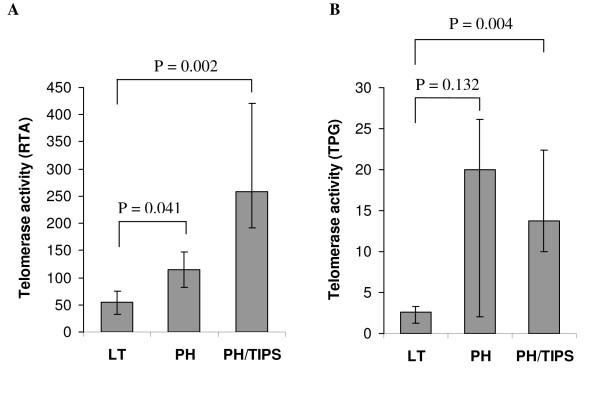
**Telomerase activity three days after surgery**. The graphs show median telomerase activity and 1st to 3rd quartile range (error bars) in liver samples from pigs three days after control laparotomy (LT, n = 6) or partial hepatectomy (PH, n = 6) with and without transjugular intrahepatic portosystemic shunt (TIPS, n = 6). Telomerase activity was determined with the real-time quantitative telomeric repeat amplification protocol (RQ-TRAP) and expressed as relative telomerase activity per μg protein (RTA; A), and by the commercially available TeloTAGGG Telomerase PCR ELISA^PLUS ^measuring telomerase activity as total product generated per μg protein (TPG; B). Median RTA values were significantly higher in the PH and PH/TIPS group compared to the LT group (Mann-Whitney U test, P < 0.05 considered significant).

We quantitated telomerase activity using a SYBR Green-based real-time quantitative telomeric repeat amplification protocol (RQ-TRAP) [25]. Sufficient tissue quality (RNA integrity) was confirmed for all liver samples by denaturing agarose gel electrophoresis and ethidium bromide staining. In contrast to a previous study showing detectable telomerase activity in a conventional end-point TRAP assay only in male liver tissue [20], we detected low telomerase activity in control liver samples (normal liver tissue without mitotic hepatocytes) of both genders without significant difference between male (n = 6) and female pigs (n = 6). Furthermore, liver samples from pigs with and without LT displayed similar telomerase activity. The median relative telomerase activity (RTA), *i.e*., telomerase activity per μg total protein in comparison to 293T cell standards, in control animals before LT was 42.2 [24.6 – 63.2] (n = 6) and not significantly different from 54.8 [32.4 – 106.4] (n = 6) in animals three days after LT (P = 0.485). Comparing median RTA levels in the three experimental groups (Figure 3A), significantly higher values were observed in the PH (115.2 [68.4 – 368.8], n = 6) and PH/TIPS group (257.8 [190.7 – 579.0], n = 6) compared to LT animals (54.8 [32.4 – 106.4], n = 6; P = 0.041 and P = 0.002, respectively). Interestingly, the animal in the LT group with mitotic hepatocytes (MI = 7) displayed a low RTA level of 40.0, whereas the animal in the PH/TIPS group without mitotic hepatocytes (MI = 0) showed an increased RTA level of 211.1. Additionally, in the PH group, the three animals without detectable mitotic hepatocytes (MI = 0) expressed elevated RTA levels [68.4 – 147.0] in comparison to the median RTA of the LT group. In summary, telomerase activity was generally increased in treated animals (PH or PH/TIPS) in comparison to control animals (LT), but with much higher RTA values in animals with detectable mitotic hepatocytes.

To confirm telomerase activation in the resection groups, we used the commercially available TeloTAGGG Telomerase PCR ELISA^PLUS ^as additional assay. This end-point assay bears limitations in reliably measuring telomerase activity in samples with high activity [25]. However, the assay includes an internal PCR control to rule out false-negative results and is thus suitable to further substantiate our findings. All samples included in this study displayed sufficient amplification of the internal PCR control, verifying absence of interference by tissue inhibitors of polymerase. In addition, telomerase activity levels, expressed as total product generated (TPG) per μg total protein in comparison to a standard with known amount of telomeric repeats (Figure 3B), were higher in PH animals (20.2 [1.7 – 42.4], n = 6) and PH/TIPS animals (13.8 [5.6 – 50.0], n = 6) in comparison to LT animals (2.6 [0.1 – 9.0], n = 6).

**Figure 4:**
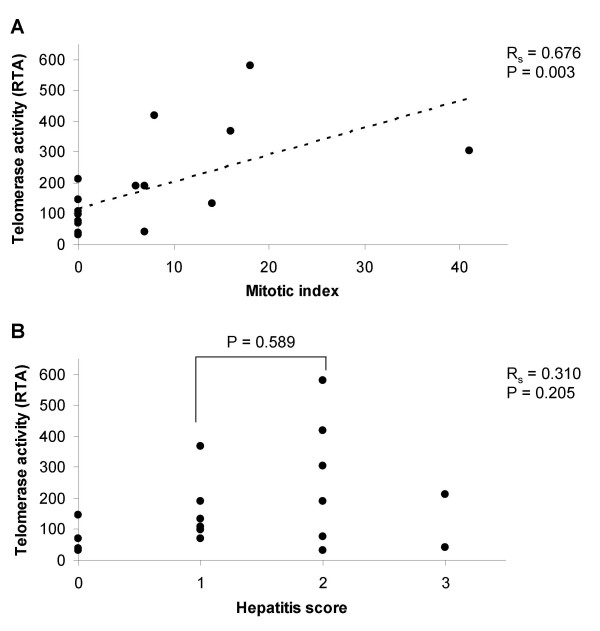
**Correlation of mitotic indices and hepatitis scores to telomerase activity**. Mitotic indices (A; n = 17) and hepatitis scores (B; n = 17) were correlated to the relative telomerase activity per μg protein (RTA) in x-y plots by adding linear trendlines. Association was tested with the Spearman rank correlation (R_S_) considering P < 0.05 significant. Histological assessment is not available for one PH animal. In a subgroup analysis, RTA levels in animals with hepatitis score 1 were compared to animals with hepatitis score 2 (Mann-Whitney U test, P < 0.05 considered significant).

Correlation of RTA levels to MI (Figure 4A) showed a significant relationship (P = 0.003) and linear association (R_s _= 0.676) with higher RTA values in liver samples with high mitotic activity. In contrary, we could not identify a correlation between RTA levels and hepatitis scores (Figure 4B). Additionally, a subgroup analysis comparing animals with hepatitis score 2 (n = 6) to animals with hepatitis score 1 (n = 6) did not reveal a significant difference in RTA levels (P = 0.589; Figure 4B). Even omitting the two animals with hepatitis score 3, did not result in a significant correlation between inflammatory infiltration and RTA levels (P = 0.158, R_s _= 0.380).

## Discussion

To investigate the association between liver regeneration with hepatocyte proliferation and telomerase activation, we studied a large animal model with telomere biology comparable to humans [20-22], and therefore, with higher relevance to human telomere biology than the commonly employed inbred mouse models. We performed 70 to 80% liver resection with and without hepatic decompression to induce compensatory hyperplasia, characterized by proliferation of normally quiescent hepatocytes [1,2]. Currently, there is no antibody available to reliably detect telomerase in tissue sections [26]. Thus, we evaluated telomerase activity employing a fully quantitative PCR-based assay. This method constitutes an alternative and well accepted approach to investigate telomerase expression in tissue samples. Based on observations in liver transplant recipients and patients with massive liver resection, hepatic decompression was performed by TIPS placement as additional procedure in a separate experimental group to improve survival and to further enhance liver regeneration [27,28]. This approach was based on the hypothesis that abrogation of increased portal pressure and shear stress following liver resection would prevent continuous liver damage and promote regeneration. Indeed, we observed higher mitotic indices in PH/TIPS animals in comparison to PH alone. Correspondingly, we also detected higher telomerase activity levels following PH/TIPS.

In our study, telomerase activity generally increased in treated animals (PH and PH/TIPS) relative to control animals (LT) with peak values in animals with high mitotic activity. Mitotic hepatocytes as marker for compensatory hyperplasia were not detected in 4 out of 11 animals three days after PH or PH/TIPS (histological assessment not available for one PH animal). Counting mitotic hepatocytes is the most direct tissue-based marker for hepatocyte proliferation, however, not sensitive enough to fully quantitate proliferative activity in regenerating liver tissue. Furthermore, mitosis constitutes a very short segment of the cell cycle and thus mitotic figures might not be present in some of the treated animals despite liver regeneration. Other proliferation markers, including immunohistochemical staining for cell cycle-associated nuclear antigens or gene expression patterns, might prove to be more reliable in future studies [29].

Our data demonstrating telomerase activation in regenerating pig liver tissue are consistent with previous findings in patients with chronic hepatitis showing *TERT *expression and telomerase activation in regenerative nodules [30,31]. Since high levels of telomerase activity have also been detected in leucocytes, it has thus far remained an area of considerable controversy whether increased levels of telomerase activity in liver samples from patients with hepatitis result from telomerase activation in hepatocytes or from infiltrating leucocytes [32]. Based on our results, we propose that telomerase activation in proliferating hepatocytes is the main cause for increased telomerase activity in regenerative liver nodules. This conclusion is supported by the significant correlation of telomerase activity to the number of mitotic hepatocytes in our study. In contrast, no relationship was observed between telomerase activity and hepatitis scores. For example, animals with control laparotomy displayed higher hepatitis scores but lower telomerase activity in comparison to animals with liver resection. Furthermore, a subgroup analysis comparing animals with grade 1 to animals with grade 2 hepatitis did not reveal a significant difference in telomerase activity.

The promoter of the human *TERT *gene, the catalytic and rate limiting component of the telomerase complex, contains transcriptional activation sites that are characteristic of many growth-related genes [33,34]. As other groups have reported, partial hepatectomy induces a rapid but transient activation of mitogenic signal transduction pathways, in particular phosphoinositide 3-kinase and mitogen-activated protein kinase/extracellular signal-regulated kinase [35]. Downstream targets of these pathways include transcription factors that activate *TERT*, for example c-Myc. Furthermore, as shown in primary mouse hepatocytes in vitro [17], major growth factors driving liver regeneration, namely hepatocyte growth factor and epidermal growth factor, are associated with telomerase activation. Pretreatment with these growth factors before partial hepatectomy increased telomerase activation in the remnant liver [17]. Together, these publications further support our data and suggest a molecular link between telomerase activation and growth factors as well as signal transduction pathways driving liver regeneration. Further investigations in animal models, such as introduced in this study, have to be conducted to elucidate the molecular mechanism of *TERT *regulation in liver regeneration. However, since the sequence of the porcine *TERT *promoter has not been reported and therefore differences in the molecular regulation of *TERT *between human and porcine hepatocytes cannot be excluded, results have to be confirmed in human liver samples and primary human hepatocyte cultures.

In cell culture, expansion of proliferating human hepatocytes is limited by replication-associated telomere erosion [12,36]. Observations in telomerase knockout mice with short telomeres suggest that telomere dysfunction inhibits liver cells with critically short telomeres from entering the cell cycle [37]. Therefore, telomere dysfunction in proliferating hepatocytes has been proposed as cellular mechanism underlying impaired regeneration in chronic liver injury [38]. In agreement with these studies and based on our findings, we suggest that telomerase activation in proliferating hepatocytes during liver regeneration represents, in analogy to what has been observed in human B lymphocytes, a protective mechanism to prevent chromosomal instability and early replicative exhaustion. Furthermore, in addition to telomere length maintenance, telomerase stabilizes telomeric ends and improves capping function, which may lead to increased cellular resistance against a wide variety of stressors and cytotoxic agents. To this regard, it has been shown that embryonic stem cells with high levels of telomerase activity accumulate lower concentrations of peroxides, implying greater resistance against oxidative stress [10]. Although, the mechanistic basis for some of these observations is not completely understood, we speculate that telomerase activation in liver regeneration might function as survival-promoting factor. Our study was not designed to investigate the functional role of telomerase activation in liver regeneration.

Recent studies suggest that permissiveness of hepatocytes for telomerase activation might be impaired during chronic hepatitis. A recent publication described absence of telomerase activation following partial hepatectomy in hepatitis B virus X gene transgenic mice [39]. In addition, transforming growth factor beta (TGF-β), a profibrogenic growth factor released during liver inflammation, rapidly represses *TERT *transcription in normal and neoplastic cells on a mechanism depending on the intracellular signaling protein Smad3 [40]. Therefore, inhibition of telomerase activation and/or accelerated telomere shortening in patients with chronic hepatitis may limit hepatocyte proliferation and survival with impairment of regenerative capability. In future experimentation, the model established in this study may be helpful to investigate consequences of telomerase inhibition on telomere dynamics in hepatocyte proliferation and liver regeneration. Such studies should be conducted before telomerase inhibitors are utilized in clinical studies to treat cancer patients [41].

## Conclusion

Our data demonstrate telomerase activation in liver regeneration by compensatory hyperplasia in a large animal model with telomere biology comparable to humans. A significant correlation between telomerase activation and the number of mitotic hepatocytes was observed, whereas, no relationship was detected between telomerase activation and the degree of inflammatory infiltration. Telomerase activation may constitute a mechanism to protect proliferating liver cells against telomere shortening and oxidative stress.

## Methods

### Animals and surgical procedures

All animals received humane care and experimentation was approved by the local review board. PH with and without TIPS or control LT were performed in 1 year old Göttinger minipigs (Ellegaard, Dalmose, Danmark). Animals evaluated in each group as well as gender and weight distribution are summarized in Table 1. All operations were performed under general anesthesia. In brief, following induction by an intramuscular injection of 5% ketamin (0.5 ml per kg body weight) and 0.1 ml Stresnil (Janssen-Cilag, Neuss, Germany) per kg body weight, an indwelling catheter was placed into the right internal jugular vein. Before endotracheal intubation, a propofol bolus (30 to 50 mg) was given. Animals were ventilated with a rate of 12 to 15 per minute and a volume of 400 to 500 ml. Anesthesia was maintained by continuous infusion of propofol (15 mg per kg body weight per hour). Animals received fentanyl (0.010 mg per kg body weight per hour) for analgesia and repeated doses of pancuroniumbromid (0.1 ml per kg body weight) for muscular relaxation. Oxygenation, heart rate, and blood pressure were monitored. The abdomen was opened and the liver mobilized. Approximately 70 to 80% of liver tissue was removed by resection of the right medial, left lateral, and left medial lobe using the clamp and finger fracture technique. Finally, blood vessels and bile duct for the remaining left medial lobe were checked for integrity and the abdomen was closed by multi-layer suture. In the PH/TIPS group, a 43 mm long and 6 mm wide Easy Wallstent (Boston Scientific, Natick, USA) was placed as TIPS via 9-French guide catheter through the right internal jugular vein under radiographic control. After extubation, intravenous fluids were given and the animals were kept for 24 hours in individual boxes with continuous warming at 26°C. Metamizol and flunixin were used for pain control. Three days after surgery, a second laparotomy was performed. Liver tissue samples were obtained and snap-frozen in liquid nitrogen and fixed in paraformaldehyde. Animals were finally euthanized by intravenous injection of 20 ml embutramid.

### Clinical chemistry

Before laparotomy, 24, 48, and 72 hours postoperatively, blood samples were obtained from each animal via indwelling jugular vein catheter. Without knowledge of the experimental groups, serum levels of albumin, bilirubin, ALT, AST, γ-glutamyl transferase, alkaline phosphatase, cholinesterase, and blood coagulation were measured. All clinical chemistry tests were performed using standard kits and reagents.

### Histological assessment

Fixed tissue samples were embedded in paraffin, sectioned (5 μm), and stained with hematoxylin and eosin. Liver histology was evaluated by a pathologist without knowledge of the experimental groups. For each animal, mitotic hepatocytes were counted in ten high-power fields (total magnification 400×) and results were recorded as MI (number of mitotic hepatocytes per ten high-power fields). Inflammatory activity (hepatitis score) was graded according to Desmet and colleagues [42] in low-power magnifications (100×) from 0 (none) to 4 (strong).

### Quantification of telomerase activity

Telomerase was extracted from each snap-frozen tissue sample following standard protocols [8]. After protein quantification with the BCA Compat-Able Protein Assay Kit (Perbio Science, Bonn, Germany), diluted extracts were used to measure telomerase activity using a fully quantitative SYBR Green-based RQ-TRAP with 293T cells as standards to calculate the RTA per μg of protein for each sample [25]. Briefly, 20 ng of protein were incubated with 160 ng telomerase primer TS, 80 ng anchored return primer ACX [43], and Universal SYBR Green PCR Master Mix (Applied Biosystems, Foster City, USA), in a final volume of 40 μl for 20 minutes at 25°C. Using the ABI Prism 7900 thermal cycler (Applied Biosystems), amplification was performed in 40 PCR cycles (30 seconds 95°C and 60 seconds 60°C). As described before [25], semi-log amplification curves were then compared with amplification plots generated from serial dilutions of telomerase-positive 293T cell extracts as standards (equivalent to 1000, 500, 100, 50, and 10 cells, respectively) and RNase-inactivated samples as negative controls.

A commercially available telomerase activity assay, the TeloTAGGG Telomerase PCR ELISA^PLUS ^(Roche Diagnostics, Mannheim, Germany), was used as additional method to confirm telomerase activity. This end-point assay utilizes an enzyme-linked immunosorbent assay for quantification and an internal PCR control to detect false-negative results due to tissue inhibitors of polymerase. The test was performed with 1000 ng protein and an initial telomerase incubation time of 20 minutes. The results were expressed as TPG per μg protein in comparison to a control template of 0.1 amole telomeric repeats [36].

### Statistical analysis

Data are presented as median [range]. Because of the small data set and ordinal hepatitis score, a two-tailed Mann-Whitney U test was employed as non-parametric test to determine significant differences between groups. Association between two variables was tested with the Spearman rank correlation test (R_S_). P < 0.05 was considered significant.

## List of abbreviations

ALT – alanine aminotransferase; AST – aspartate aminotransferase; LT – laparotomy; MI – mitotic index; PH – partial hepatectomy; RQ-TRAP – real-time quantitative telomeric repeat amplification protocol; RTA – relative telomerase activity; TIPS – transjugular intrahepatic portosystemic shunt; TPG – total product generated

## Competing interests

The author(s) declare that they have no competing interest.

## Authors' contributions

HW conceived the design of the study and carried out telomerase quantification and statistical evaluations. Surgical procedures and sample collection were performed by AM, LM and CH. Histological assessment was provided by SP. JP participated in conceiving the design of the study and drafting the paper. All authors read and approved the paper.

## References

[B1] Columbano A, Shinozuka H (1996). Liver regeneration versus direct hyperplasia. FASEB J.

[B2] Fausto N, Campbell JS, Riehle KJ (2006). Liver regeneration. Hepatology.

[B3] Fausto N (2004). Liver regeneration and repair: hepatocytes, progenitor cells, and stem cells. Hepatology.

[B4] Roskams TA, Libbrecht L, Desmet VJ (2003). Progenitor cells in diseased human liver. Semin Liver Dis.

[B5] Blackburn EH (2000). Telomere states and cell fates. Nature.

[B6] Collins K (2000). Mammalian telomeres and telomerase. Curr Opin Cell Biol.

[B7] Shay JW, Pereira-Smith OM, Wright WE (1991). A role for both RB and p53 in the regulation of human cellular senescence. Exp Cell Res.

[B8] Kim NW, Piatyszek MA, Prowse KR, Harley CB, West MD, Ho PL, Coviello GM, Wright WE, Weinrich SL, Shay JW (1994). Specific association of human telomerase activity with immortal cells and cancer. Science.

[B9] Greider CW, Blackburn EH (1985). Identification of a specific telomere terminal transferase activity in Tetrahymena extracts. Cell.

[B10] Armstrong L, Saretzki G, Peters H, Wappler I, Evans J, Hole N, von Zglinicki T, Lako M (2005). Overexpression of telomerase confers growth advantage, stress resistance, and enhanced differentiation of ESCs toward the hematopoietic lineage. Stem Cells.

[B11] Lee MK, Hande MP, Sabapathy K (2005). Ectopic mTERT expression in mouse embryonic stem cells does not affect differentiation but confers resistance to differentiation- and stress-induced p53-dependent apoptosis. J Cell Sci.

[B12] Wege H, Chui MS, Le HT, Strom SC, Zern MA (2003). In vitro expansion of human hepatocytes is restricted by telomere-dependent replicative aging. Cell Transplant.

[B13] Harle-Bachor C, Boukamp P (1996). Telomerase activity in the regenerative basal layer of the epidermis inhuman skin and in immortal and carcinoma-derived skin keratinocytes. Proc Natl Acad Sci U S A.

[B14] Hu BT, Insel RA (1999). Up-regulation of telomerase in human B lymphocytes occurs independently of cellular proliferation and with expression of the telomerase catalytic subunit. Eur J Immunol.

[B15] Martens UM, Brass V, Sedlacek L, Pantic M, Exner C, Guo Y, Engelhardt M, Lansdorp PM, Waller CF, Lange W (2002). Telomere maintenance in human B lymphocytes. Br J Haematol.

[B16] Takagi S, Kinouchi Y, Chida M, Hiwatashi N, Noguchi M, Takahashi S, Shimosegawa T (2003). Strong telomerase activity of B lymphocyte from mesenteric lymph nodes of patients with inflammatory bowel disease. Dig Dis Sci.

[B17] Inui T, Shinomiya N, Fukasawa M, Kobayashi M, Kuranaga N, Ohkura S, Seki S (2002). Growth-related signaling regulates activation of telomerase in regenerating hepatocytes. Exp Cell Res.

[B18] Ritz JM, Kuhle O, Riethdorf S, Sipos B, Deppert W, Englert C, Gunes C (2005). A novel transgenic mouse model reveals humanlike regulation of an 8-kbp human TERT gene promoter fragment in normal and tumor tissues. Cancer Res.

[B19] Horikawa I, Chiang YJ, Patterson T, Feigenbaum L, Leem SH, Michishita E, Larionov V, Hodes RJ, Barrett JC (2005). Differential cis-regulation of human versus mouse TERT gene expression in vivo: identification of a human-specific repressive element. Proc Natl Acad Sci U S A.

[B20] Fradiani PA, Ascenzioni F, Lavitrano M, Donini P (2004). Telomeres and telomerase activity in pig tissues. Biochimie.

[B21] Jeon HY, Hyun SH, Lee GS, Kim HS, Kim S, Jeong YW, Kang SK, Lee BC, Han JY, Ahn C, Hwang WS (2005). The analysis of telomere length and telomerase activity in cloned pigs and cows. Mol Reprod Dev.

[B22] Kozik A, Bradbury EM, Zalensky A (1998). Increased telomere size in sperm cells of mammals with long terminal (TTAGGG)n arrays. Mol Reprod Dev.

[B23] Russo V, Berardinelli P, Martelli A, Di GO, Nardinocchi D, Fantasia D, Barboni B (2006). Expression of telomerase reverse transcriptase subunit (TERT) and telomere sizing in pig ovarian follicles. J Histochem Cytochem.

[B24] Kahn D, Hickman R, Terblanche J, von Sommoggy S (1988). Partial hepatectomy and liver regeneration in pigs--the response to different resection sizes. J Surg Res.

[B25] Wege H, Chui MS, Le HT, Tran JM, Zern MA (2003). SYBR Green real-time telomeric repeat amplification protocol for the rapid quantification of telomerase activity. Nucleic Acids Res.

[B26] Wu YL, Dudognon C, Nguyen E, Hillion J, Pendino F, Tarkanyi I, Aradi J, Lanotte M, Tong JH, Chen GQ, Segal-Bendirdjian E (2006). Immunodetection of human telomerase reverse-transcriptase (hTERT) re-appraised: nucleolin and telomerase cross paths. J Cell Sci.

[B27] Koyama S, Sato Y, Hatakeyama K (2003). The subcutaneous splenic transposition prevents liver injury induced by excessive portal pressure after massive hepatectomy. Hepatogastroenterology.

[B28] Sugimoto H, Kaneko T, Hirota M, Nagasaka T, Kobayashi T, Inoue S, Takeda S, Kiuchi T, Nakao A (2004). Critical progressive small-graft injury caused by intrasinusoidal pressure elevation following living donor liver transplantation. Transplant Proc.

[B29] Assy N, Minuk GY (1997). Liver regeneration: methods for monitoring and their applications. J Hepatol.

[B30] Hytiroglou P, Kotoula V, Thung SN, Tsokos M, Fiel MI, Papadimitriou CS (1998). Telomerase activity in precancerous hepatic nodules. Cancer.

[B31] Kotoula V, Hytiroglou P, Pyrpasopoulou A, Saxena R, Thung SN, Papadimitriou CS (2002). Expression of human telomerase reverse transcriptase in regenerative and precancerous lesions of cirrhotic livers. Liver.

[B32] Lechel A, Manns MP, Rudolph KL (2004). Telomeres and telomerase: new targets for the treatment of liver cirrhosis and hepatocellular carcinoma. J Hepatol.

[B33] Ducrest AL, Szutorisz H, Lingner J, Nabholz M (2002). Regulation of the human telomerase reverse transcriptase gene. Oncogene.

[B34] Kyo S, Inoue M (2002). Complex regulatory mechanisms of telomerase activity in normal and cancer cells: how can we apply them for cancer therapy?. Oncogene.

[B35] Coutant A, Rescan C, Gilot D, Loyer P, Guguen-Guillouzo C, Baffet G (2002). PI3K-FRAP/mTOR pathway is critical for hepatocyte proliferation whereas MEK/ERK supports both proliferation and survival. Hepatology.

[B36] Wege H, Le HT, Chui MS, Liu L, Wu J, Giri R, Malhi H, Sappal BS, Kumaran V, Gupta S, Zern MA (2003). Telomerase reconstitution immortalizes human fetal hepatocytes without disrupting their differentiation potential. Gastroenterology.

[B37] Satyanarayana A, Wiemann SU, Buer J, Lauber J, Dittmar KE, Wustefeld T, Blasco MA, Manns MP, Rudolph KL (2003). Telomere shortening impairs organ regeneration by inhibiting cell cycle re-entry of a subpopulation of cells. EMBO J.

[B38] Satyanarayana A, Manns MP, Rudolph KL (2004). Telomeres and telomerase: a dual role in hepatocarcinogenesis. Hepatology.

[B39] Kojima H, Kaita KD, Xu Z, Ou JH, Gong Y, Zhang M, Minuk GY (2003). The absence of up-regulation of telomerase activity during regeneration after partial hepatectomy in hepatitis B virus X gene transgenic mice. J Hepatol.

[B40] Li H, Xu D, Li J, Berndt MC, Liu JP (2006). Transforming growth factor beta suppresses human telomerase reverse transcriptase by Smad3 interactions with C-Myc and hTERT gene. J Biol Chem.

[B41] Shay JW, Wright WE (2006). Telomerase therapeutics for cancer: challenges and new directions. Nat Rev Drug Discov.

[B42] Desmet VJ, Gerber M, Hoofnagle JH, Manns M, Scheuer PJ (1994). Classification of chronic hepatitis: diagnosis, grading and staging. Hepatology.

[B43] Kim NW, Wu F (1997). Advances in quantification and characterization of telomerase activity by the telomeric repeat amplification protocol (TRAP). Nucleic Acids Res.

